# Supplementary material for: Tear matrix metalloproteinase-9 levels may help to follow a ocular surface injury in lagophthalmic eyes
Source: PLoS One. 2022 Sep 9;17(9):e0274173. doi: 10.1371/journal.pone.0274173 (PMC9462780; doi:10.1371/journal.pone.0274173)
Supplement: Attachment — Submitted filename: Mahelkova Response to Reviewers.docx [file pone.0274173.s001.docx]

Dear Reviewer #1,

Thank you for your comments on our manuscript *"Tear matrix metalloproteinase-9 levels may help to follow a ocular surface injury in lagophthalmic eyes"*.

We have gone through them carefully and revised the manuscript accordingly.

We supplemented the discussion detailing the effect of patient’s compliance with therapy on the ocular surface (originally lines 234-237). As per the recommendation we have replaced the use of paired t-test with Wilcoxon singed-rank test. We have also included a Conclusions section.

We agree that many articles have been published already, which suggests that measurement of ocular surface MMP-9 levels can be a useful marker in revealing ocular inflammation. Rather, our study aimed to test the usefulness of this approach in a specific group of patients with lagophthalmos.

Dear Reviewer #2,

Thank you for your comments on our manuscript *"Tear matrix metalloproteinase-9 levels may help to follow a ocular surface injury in lagophthalmic eyes"*.

We have gone through them carefully and revised the manuscript accordingly.

We have clarified the name of the test – Oxford Scheme for Ocular Surface Fluorescein Staining test.

We replaced the format of the results and present them now as mean ± SEM.

We added the information about the results format to the beginning of Results section.

We have supplemented the discussion on the considerable variance in MMP-9 values. We have also added a reference (No.35).

Dear Academic Editor,

Thank you for your comments on our manuscript *"Tear matrix metalloproteinase-9 levels may help to follow a ocular surface injury in lagophthalmic eyes"*.

1. We have gone through them carefully and revised the manuscript accordingly.We verified the manuscript to meet PLOS ONE’s style requirements.
2. We added the information regarding the form of informed consent.
3. We apologize for placing the funding declaration improperly. We removed it from the Acknowledgements section and included the statement in the cover letter. Thank you for informing us and suggesting the correction.
4. We also included the Data Availability statement within the cover letter. The minimal dataset underlying our results described in our manuscript is presented within Tables 1 and 2 of the manuscript.
5. We reviewed the manuscript reference list. According to Reviewer 2 comments we added a reference (No. 35).
